# Genome-wide association reveals genetic effects on human Aβ_42 _and τ protein levels in cerebrospinal fluids: a case control study

**DOI:** 10.1186/1471-2377-10-90

**Published:** 2010-10-08

**Authors:** Mi-Ryung Han, Gerard D Schellenberg, Li-San Wang

**Affiliations:** 1 Genomics and Computational Biology Program, University of Pennsylvania, Philadelphia, PA 19104, USA; 2 Department of Pathology and Laboratory Medicine, University of Pennsylvania, Philadelphia, PA 19104, USA; 3 Institute on Aging, University of Pennsylvania, Philadelphia, PA 19104, USA; 4 Penn Center for Bioinformatics, University of Pennsylvania, Philadelphia, PA 19104, USA

## Abstract

**Background:**

Alzheimer's disease (AD) is common and highly heritable with many genes and gene variants associated with AD in one or more studies, including APOE ε2/ε3/ε4. However, the genetic backgrounds for normal cognition, mild cognitive impairment (MCI) and AD in terms of changes in cerebrospinal fluid (CSF) levels of Aβ_1-42_, T-tau, and P-tau_181P_, have not been clearly delineated. We carried out a genome-wide association study (GWAS) in order to better define the genetic backgrounds to these three states in relation to CSF levels.

**Methods:**

Subjects were participants in the Alzheimer's Disease Neuroimaging Initiative (ADNI). The GWAS dataset consisted of 818 participants (mainly Caucasian) genotyped using the Illumina Human Genome 610 Quad BeadChips. This sample included 410 subjects (119 Normal, 115 MCI and 176 AD) with measurements of CSF Aβ_1-42_, T-tau, and P-tau_181P _Levels. We used PLINK to find genetic associations with the three CSF biomarker levels. Association of each of the 498,205 SNPs was tested using additive, dominant, and general association models while considering APOE genotype and age. Finally, an effort was made to better identify relevant biochemical pathways for associated genes using the ALIGATOR software.

**Results:**

We found that there were some associations with APOE genotype although CSF levels were about the same for each subject group; CSF Aβ_1-42 _levels decreased with APOE gene dose for each subject group. T-tau levels tended to be higher among AD cases than among normal subjects. From adjusted result using APOE genotype and age as covariates, no SNP was associated with CSF levels among AD subjects. *CYP19A1 *'aromatase' (rs2899472), *NCAM2*, and multiple SNPs located on chromosome 10 near the *ARL5B *gene demonstrated the strongest associations with Aβ_1-42 _in normal subjects. Two genes found to be near the top SNPs, *CYP19A1 *(rs2899472, p = 1.90 × 10^-7^) and *NCAM2 *(rs1022442, p = 2.75 × 10^-7^) have been reported as genetic factors related to the progression of AD from previous studies. In AD subjects, APOE ε2/ε3 and ε2/ε4 genotypes were associated with elevated T-tau levels and ε4/ε4 genotype was associated with elevated T-tau and P-tau_181P _levels. Pathway analysis detected several biological pathways implicated in Normal with CSF β-amyloid peptide (Aβ_1-42_).

**Conclusions:**

Our genome-wide association analysis identified several SNPs as important factors for CSF biomarker. We also provide new evidence for additional candidate genetic risk factors from pathway analysis that can be tested in further studies.

## Background

Alzheimer's disease (AD) is the most common cause of dementia and the most prevalent neurodegenerative disorder. An estimated 10 percent of Americans over the age of 65 and half of those over age 85 have AD. More than 4.5 million Americans currently suffer from the disease. In autosomal dominant early-onset Alzheimer's disease (EOAD, age of onset < 60 years), three susceptible genes (*APP*, *PSEN1*, and *PSEN2*) have been identified [1,2]. Late-onset AD (LOAD) has ~80% heritability, and is strongly associated with apolipoprotein E (*APOE*) [3]. APOE has three major alleles (ε2/ε3/ε4) that have different effects on the risk of LOAD, with ε4 having between 10 and 30 times of risk of developing AD by 75 years of age [4].

In addition, several genetic studies have identified putative susceptible loci and genetic variants, including sortilin-related receptor (*SORL1*) [5,6], death-associated protein kinase 1 (*DAPK1*) [7], ubiquilin 1 (*UBQLN1*) [8], adenosine triphosphate-binding cassette transporter 1, subfamily A (*ABCA1*) [9], and low-density lipoprotein receptor-related protein 6 (*LRP6*) [10]. Besides these findings, a large meta-analysis from the AlzGene database [11] reported 598 potential AD-susceptibility genes. For the past few years, genome-wide genotyping association studies brought considerable success by reporting new susceptible loci for AD such as Golgi membrane protein 1 (*GOLM1*) [12-15]. Recently two groups published the two largest LOAD GWAS [16,17]; Harold *et al*. [16] reported the association of SNPs in clusterin (*CLU*) and phosphatidylinositol binding clathrin assembly protein (*PICALM*), and Lambert *et al*. [17] reported association of clusterin (*CLU*) with LOAD and additionally reported a novel association with complement component (3b/4b) receptor 1 (*CR1*). These new findings have provided valuable insights in the genetics, neuropathologic mechanisms and pathways associated with AD.

The cerebrospinal fluid (CSF) components β-amyloid peptide (Aβ_1-42_), total tau protein (T-tau) and phosphorylated tau (P-tau_181P_) are biomarkers for AD and can be used to aid in diagnosis and to predict progression from mild cognitive impairment (MCI) to AD [18,19]. These biomarkers can potentially be used in future applications to predict the development of MCI in cognitively normal subjects, progression to AD in MCI patients, and to monitor AD progression [20-23]. These biomarkers may also be used to reveal genes that are important in AD pathogenesis. In the present study, we assessed the several putative AD genes associated with CSF biomarkers that were identified from major public GWAS dataset for Alzheimer's disease, the Alzheimer's disease Neuroimaging Initiative (ADNI). This initiative is the most comprehensive effort to identify neuroimaging measures and biomarkers associated with cognitive and functional changes in healthy elderly people and in people who have MCI and AD [24]. The ADNI data is useful for researchers who are searching for genes that contribute to the development of Alzheimer's disease, which currently affects more than 4.5 million people in the United States alone. We also investigated possible associations of CSF biomarkers (Aβ_1-42_, T-tau, and P-tau_181P_) with the number of *APOE *ε4 allele, age and *APOE *genotype in order to improve the characterization of the CSF biomarkers and genome-wide SNP genotyping data from the ADNI cohort. This is the first genome-wide association study to use these AD-related biomarkers to identify genes critical the pathogenesis of AD.

## Methods

### Sample

Data used in the preparation of this article were obtained from the Alzheimer's Disease Neuroimaging Initiative (ADNI) database http://www.loni.ucla.edu/ADNI. ADNI was launched in 2003 by the National Institute on Aging (NIA), the National Institute of Biomedical Imaging and Bioengineering (NIBIB), the Food and Drug Administration (FDA), private pharmaceutical companies and non-profit organizations, as a $60 million, 5-year public-private partnership. The primary goal of ADNI has been to test whether serial magnetic resonance imaging (MRI), positron emission tomography (PET), other biological markers, and clinical and neuropsychological assessment can be combined to measure the progression of mild cognitive impairment (MCI) and early Alzheimer's disease (AD). Determination of sensitive and specific markers of very early AD progression is intended to aid researchers and clinicians to develop new treatments and monitor their effectiveness, as well as lessen the time and cost of clinical trials.

The Principle Investigator of this initiative is Michael W. Weiner, M.D., VA Medical Center and University of California - San Francisco. ADNI is the result of efforts of many co-investigators from a broad range of academic institutions and private corporations, and subjects have been recruited from over 50 sites across the U.S. and Canada. The initial goal of ADNI was to recruit 800 adults, ages 55 to 90, to participate in the research -- approximately 200 cognitively normal older individuals to be followed for 3 years, 400 people with MCI to be followed for 3 years, and 200 people with early AD to be followed for 2 years. For up-to-date information see http://www.adni-info.org.

The GWAS dataset (downloaded from ADNI website in June 2009) consisted of 243 normal, 235 MCI and 340 AD subjects genotyped using the Illumina Human Genome 610 Quad BeadChips. 410 of these subjects (119 Normal, 115 MCI and 176 AD; 247 males and 163 females) have CSF Aβ_1-42_, T-tau, and P-tau_181P _levels. Detailed protocols for subject recruitment and biomarker accrual are available at the ADNI website http://www.adni-info.org/. Demographics, CSF biomarkers, and *APOE *genotype data of the ADNI dataset are summarized in Table 1, Table 2 and Additional file 1.

**Table 1:** Demographic, clinical and biomarker data for each subject group after removing 20 outliers (n = 390: Normal, MCI and AD)

Normal (n = 109)	Mean
Age (year)	71.7
CSF Aβ_1-42 _levels (pg/ml)	205.8
CSF P-tau_181P _levels (pg/ml)	25.4
CSF T-tau levels (pg/ml)	71.8
Last MMSE	29

	**N**

Male	59
Female	50

**MCI (n = 109)**	**Mean**

Age (year)	70
CSF Aβ_1-42 _levels (pg/ml)	170.4
CSF P-tau_181P _levels (pg/ml)	33.7
CSF T-tau level (pg/ml)	99.5
Last MMSE	26.5

	**N**

Male	72
Female	37

**AD (n = 172)**	**Mean**

Age (year)	71
CSF Aβ_1-42 _levels (pg/ml)	144.7
CSF P-tau_181P _levels (pg/ml)	40.3
CSF T-tau levels (pg/ml)	115.3
Last MMSE	21.1

	**N**

Male	105
Female	67

Abbreviations: CSF, cereb al spinal fluid; MMSE, Mini Mental State Examination

**Table 2:** *APOE *genotype data for each subject group (Normal, MCI and AD)

*APOE *genotype data (after removing outliers: n = 390)							
							
	
***APOE *genotype**		**ε2/ε2**	**ε2/ε3**	**ε2/ε4**	**ε3/ε3**	**ε3/4**	**ε4/ε4**
	
Normal (n = 109)	**n**	0	18	2	66	21	2
MCI (n = 109)	**n**	0	10	2	47	40	10
AD (n = 172)	**n**	0	1	3	54	81	33
							
***APOE *allele**		**ε2**		**ε3**		**ε4**	
	
Normal (n = 109)	**n**	20		105		25	
MCI (n = 109)	**n**	12		97		52	
AD (n = 172)	**n**	4		136		117	

							
***APOE *genotype data before removing outliers: n = 410)**							

							
	
***APOE *genotype**		**ε2/ε2**	**ε2/ε3**	**ε2/ε4**	**ε3/ε3**	**ε3/ε4**	**ε4/ε4**
	
Normal (n = 119)	**n**	0	20	2	71	24	2
MCI (n = 115)	**n**	0	10	2	52	41	10
AD (n = 176)	**n**	0	2	3	54	83	34
							
***APOE *allele**		**ε2**		**ε3**		**ε4**	
	
Normal (n = 119)	**n**	22		115		28	
MCI (n = 115)	**n**	12		103		53	
AD (n = 176)	**n**	5		139		120	

Abbreviations: CSF, cerebral spinal fluid

### Genotyping and sample quality control

Quality control for the genotyping data was performed using PLINK http://pngu.mgh.harvard.edu/~purcell/plink/[25] as follows. 498,205 SNPs were retained after excluding SNPs with Minor Allele Frequency (MAF) < 5%, call rate < 98%, or significant in Hardy-Weinberg Equilibrium test (p ≤ 10^-6^). All samples had genotyping call rate > 95% and were retained. We then examined population stratification by visual inspection using the first two dimensions from principal components analysis, using SmartPCA from EIGENSTRAT http://genepath.med.harvard.edu/~reich/Software.htm[26,27]. Self-reported ethnicity and racial identities for ADNI subjects were used to highlight samples in the PCA plot and are summarized in Additional file 2. 390 samples were retained after SmartPCA excluded 20 samples as outliers. We computed the top five principal component coordinates using SmartPCA to correct for stratification in association analysis. SmartPCA removed all but one of the Asian samples and retained Black/African Americans (Additional file 3); Visual inspection suggests that the first principal component (PC0), which explains the most variance in the data, separates the Caucasians and non-Caucasians reasonably well (setting threshold PC0 < = 0.01 can exclude all non-Caucasians). Finally, we excluded 52 non-Caucasian samples as outliers; the genomic control variance inflation factor λ was 1.00983, suggesting minimal population admixture in the final sample used for association analysis. We performed association analysis using age and APOE ε4 genotype as covariate and did not incorporate principal components. Quantile-Quantile plots for each of the three test groups with log10-transformed level of three CSF biomarkers (Additional file 4 and Additional file 5) suggested that population stratification having negligible bias on the genetic associations (Additional file 6). Finally, for the whole analysis we performed in the following method section, the study sample of 390 individuals with three CSF biomarkers was used after removing 20 outliers. The level of three CSF biomarkers was log10-transformed.

### Effect of *APOE *ε4 copy number on CSF biomarker levels

We performed Kruskal-Wallis test of log_10_-transformed CSF biomarkers (Aβ_1-42_, T-tau, and P-tau_181P_) stratified by the number of APOE ε4 alleles and the APOE genotypes across all 390 samples or within each of the three diagnostic groups.

### Association testing of SNPs and CSF analysis

We tested SNP association with the three CSF biomarkers (Aβ_1-42_, T-tau, and P-tau_181P_) by PLINK using samples and SNP markers passing QC. CSF biomarker levels were log_10_-transformed so they become normally distributed. The association analysis used a full linear model comprising three genetic effects: additive effects of allele dosage (ADD), dominance deviation from additivity (DOMDEV) (negative means the allele is recessive), and 2-df joint test of both additive and dominance (GENO_2DF). In addition, we used age (of being recruited by the study) and APOE ε4 genotype (number of APOE ε4 allele; 0, 1, 2) as covariates after removing outliers. To ensure the significance is not due to population stratification, we also incorporated the top five PCA principal components in the linear regression to further control for the population structure, but found the addition has very little effect on the statistical significance.

### Gene ontology and E-SNP analysis

We carried out gene ontology analysis of SNP association results using ALIGATOR (Association LIst Go AnnoTatOR) [28], to find gene-ontology terms enriched with significant SNPs. We used p-value cutoff < 10^-3 ^for SNPs, 5000 replicate gene lists and 1000 permutations as parameters to run ALIGATOR. We examined the top associated SNPs and examined nearby SNPs in linkage disequilibrium (LD) that are associated with gene expression from published eQTL studies [29-33].

## Results and Discussion

### Association of SNPs with CSF biomarkers

**Figure 1:**
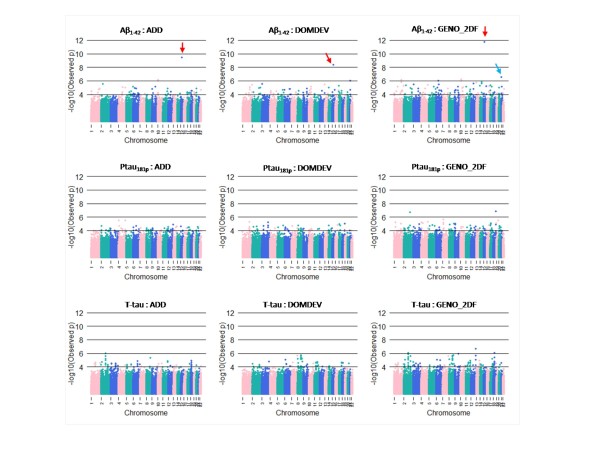
**Manhattan plots of the quantitative trait (CSF biomarkers: T-tau, P-tau_181P _and Aβ_1-42_) genome wide association analysis in normal subjects**. Colors on x-axis indicate an autosomal chromosome (from chromosome 1 to chromosome 22). The y-axis indicates p-values (-log_10_(observed p-values)). Red arrows indicate rs2899472 on chromosome 15 and blue arrow indicates rs1022442 on chromosome 21.

**Figure 2:**
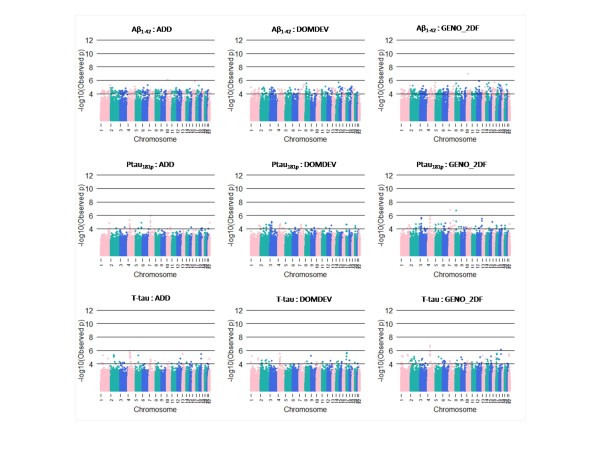
**Manhattan plots of the quantitative trait (CSF biomarkers: T-tau, P-tau_181P _and Aβ_1-42_) genome wide association analysis in MCI subjects**. Colors on x-axis indicate an autosomal chromosome (from chromosome 1 to chromosome 22). The y-axis indicates p-values (-log_10_(observed p-values)).

**Figure 3:**
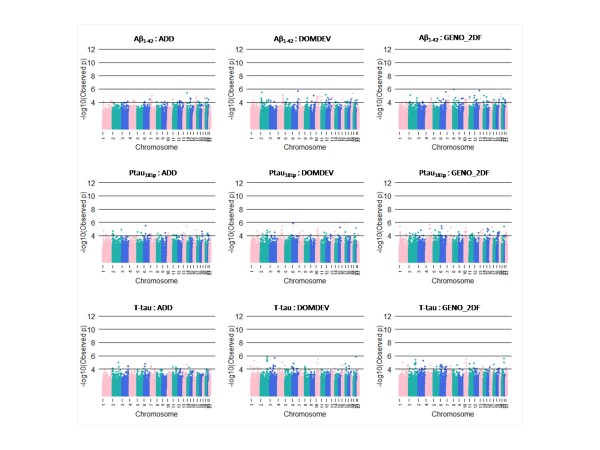
**Manhattan plots of the quantitative trait (CSF biomarkers: T-tau, P-tau_181P _and Aβ_1-42_) genome wide association analysis in AD subjects**. Colors on x-axis indicate an autosomal chromosome (from chromosome 1 to chromosome 22). The y-axis indicates p-values (-log_10_(observed p-values)).

**Figure 4:**
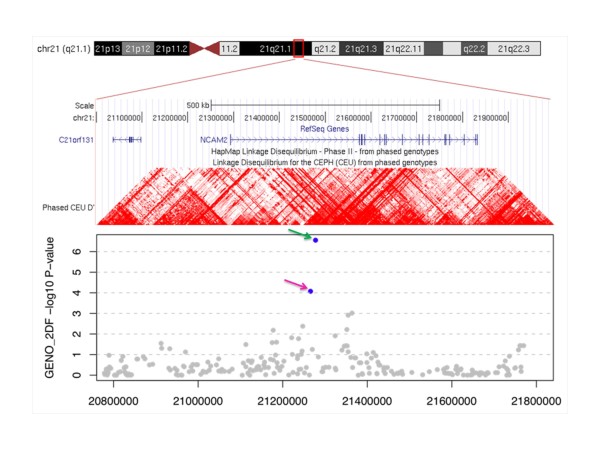
**Upper image shows genes in linkage disequilibrium with the SNP rs1022442 and lower plot shows the distribution of SNP rs1022442 and nearby SNPs**. In lower plot, the x-axis indicates loci on chromosome 21 and y-axis indicates p-values (-log_10_(observed GENO_2DF p-values)). Green arrow indicates rs1022442 (p = 2.75 × 10^-7^) on loci 21277717 and pink arrow indicates rs2826629 (p = 8.40 × 10^-5^) on loci 21265887.

**Figure 5:**
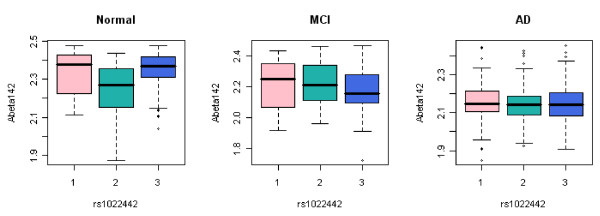
**Boxplots of Aβ_1-42 _levels in normal, MCI and AD subjects stratified by rs1022442 genotype**. The x-axis indicates AA, AB and BB respectively.

We summarize the top SNPs (p-value < 10^-6^) with and without using covariates (APOE genotype and age) in Table 3. Without using covariates, we found some genes near the top SNPs, including *CYP19A1 *(rs2899472, p = 1.86 × 10^-7^) and *TOMM40 *(rs2075650, p = 3.03 × 10^-7^) from Aβ_1-42 _in normal subject. Several genetic studies have identified those genes as putative susceptible loci and genetic variants associated with Alzheimer's disease [34,35]. However, close examination of nearby SNPs showed rs2899472 and rs2075650 were not supported by nearby SNPs in LD (nearby SNPs in LD are all non-significant). Because APOE genotypes are strongly associated with AD and TOMM40 is physically close to APOE, we focused on SNPs from adjusted results that consider APOE genotype and age. Here, we found 10 SNPs significantly associated with CSF biomarker level of Aβ_1-42_, 3 SNPs from T-tau, and 2 SNPs from P-tau_181P _at 10^-6 ^significance level in normal subjects (Figure 1). In addition, we found 1 SNP significantly associated with CSF biomarker level of Aβ_1-42_, 3 SNPs from T-tau, and 2 SNPs from P-tau_181P _at 10^-6 ^significance level in MCI subjects (Figure 2). No SNPs were found at 10^-6 ^significance level in AD subjects (Figure 3). For normal subjects, we found genes near the top SNPs, included *CYP19A1 *(rs2899472, p = 1.90 × 10^-7^), *NCAM2 *(rs1022442, p = 2.75 × 10^-7^) for Aβ_1-42 _association and *UPP2 *(rs2074955, p = 2.07 × 10^-7^) for P-tau_181p _association. Again, close examination of SNPs in LD with rs2899472 (CYP19A1) did not support rs2899472. The SNP rs1022442 was in close to genome-wide significance, supported by nearby SNPs (Figure 4), and *NCAM2 *(neural cell adhesion molecule 2) gene was reported as a genetic factor related to the progression of AD in the Japanese population [36]. The Aβ_1-42 _level grouped by the SNP rs1022442 genotype over all three cohorts (normal, MCI and AD) supports our finding (Figure 5). Boxplots of Aβ_1-42 _levels in normal subjects stratified by rs1022442 genotype showed significant differences between AA, AB and BB. Previous study indicated an increased risk associated with rs2899472 in AD patients, which was amplified in APOE ε4 carriers in their study [35]. For MCI subjects, we found several genes near the top SNPs, included *FLJ21511 *(rs2768975; p = 1.96 × 10^-7^, rs6850199; p = 3.18 × 10^-7^) by T-tau association and *CHN2 *(rs121724, p = 1.45 × 10^-7^), *MTUS1 *(rs7842088, p = 2.12 × 10^-7^) by P-tau_181p _association.

**Table 3:** Linear regression result for SNPs with CSF biomarkers in Normal, MCI and AD subjects (Top SNPs with p-value < 10^-6^)

Linear regression result without using covariates								
**Normal: Aβ**_**1-42**_								

**Chromosome**	**SNP**	**Position**	**A1**	**TEST**	**BETA**	**STAT**	**P-value**	**Gene Symbol, Gene Name**

15	rs2899472	49303347	A	GENO_2DF	NA	40.21	1.86E-09	CYP19A1, cytochrome p450, family 19, subfamily a, polypeptide 1
15	rs2899472	49303347	A	DOMDEV	0.1791	5.859	5.40E-08	CYP19A1, cytochrome p450, family 19, subfamily a, polypeptide 1
1	rs11206801	56623274	A	GENO_2DF	NA	33.14	6.37E-08	
1	rs4431886	56624855	C	GENO_2DF	NA	33.14	6.37E-08	
15	rs2899472	49303347	A	ADD	-0.1407	-5.699	1.11E-07	CYP19A1, cytochrome p450, family 19, subfamily a, polypeptide 1
19	rs2075650	50087459	G	GENO_2DF	NA	30.02	3.03E-07	TOMM40, translocase of outer mitochondrial membrane 40 homolog (yeast)
12	rs10784496	64447238	G	GENO_2DF	NA	29.98	3.09E-07	
7	rs12534221	130938530	A	GENO_2DF	NA	27.83	9.04E-07	
6	rs1727638	72196293	A	GENO_2DF	NA	27.69	9.71E-07	

**Normal: T-tau**								

**Chromosome**	**SNP**	**Position**	**A1**	**TEST**	**BETA**	**STAT**	**P-value**	**Gene Symbol, Gene Name**

12	rs1997111	117872301	T	GENO_2DF	NA	36.66	1.10E-08	
12	rs11069178	117852210	C	GENO_2DF	NA	31.23	1.65E-07	
2	rs17189298	119561787	A	GENO_2DF	NA	29.51	3.90E-07	
8	rs2935776	109699079	C	GENO_2DF	NA	28.81	5.53E-07	
8	rs2928826	109683270	T	GENO_2DF	NA	28.73	5.77E-07	
2	rs895401	119584178	G	GENO_2DF	NA	27.86	8.90E-07	
2	rs893769	119556554	C	ADD	-0.1024	-5.237	8.92E-07	

**Normal: P-tau**_**181p**_								

**Chromosome**	**SNP**	**Position**	**A1**	**TEST**	**BETA**	**STAT**	**P-value**	**Gene Symbol, Gene Name**

18	rs1943816	69209602	C	GENO_2DF	NA	32.09	1.08E-07	
2	rs2074955	158686040	C	GENO_2DF	NA	32.06	1.09E-07	UPP2, uridine phosphorylase 2

**MCI: T-tau**								

**Chromosome**	**SNP**	**Position**	**A1**	**TEST**	**BETA**	**STAT**	**P-value**	**Gene Symbol, Gene Name**

22	rs5998432	31075916	T	GENO_2DF	NA	27.91	8.696E-07	

**MCI: P-tau**_**181p**_								

**Chromosome**	**SNP**	**Position**	**A1**	**TEST**	**BETA**	**STAT**	**P-value**	**Gene Symbol, Gene Name**

2	rs7558386	227270383	G	GENO_2DF	NA	32.09	1.08E-07	
2	rs7558386	227270383	G	DOMDEV	0.242	5.343	5.27E-07	
3	rs7631605	37209593	T	GENO_2DF	NA	27.71	9.60E-07	

**AD: Aβ**_**1-42**_								

**Chromosome**	**SNP**	**Position**	**A1**	**TEST**	**BETA**	**STAT**	**P-value**	**Gene Symbol, Gene Name**

21	rs239713	27618347	T	GENO_2DF	NA	29.03	4.96E-07	

**AD: T-tau**								

**Chromosome**	**SNP**	**Position**	**A1**	**TEST**	**BETA**	**STAT**	**P-value**	**Gene Symbol, Gene Name**

20	rs4925189	59380439	G	DOMDEV	0.1969	5.178	6.35E-07	CDH4, cadherin 4, type 1, R-cadherin (retinal)

**AD: P-tau**_**181p**_								

**Chromosome**	**SNP**	**Position**	**A1**	**TEST**	**BETA**	**STAT**	**P-value**	**Gene Symbol, Gene Name**

4	rs12643654	96378840	G	GENO_2DF	NA	29.04	4.95E-07	UNC5C, unc-5 homolog C (C. elegans)

**Linear regression result using APOE genotype and age as covariates**								

**Normal: Aβ**_**1-42**_								

**Chromosome**	**SNP**	**Position**	**A1**	**TEST**	**BETA**	**STAT**	**P-value**	**Gene Symbol, Gene Name**

15	rs2899472	49303347	A	GENO_2DF	NA	53.98	1.90E-12	CYP19A1, cytochrome p450, family 19, subfamily a, polypeptide 1
15	rs2899472	49303347	A	ADD	-0.1393	-6.925	3.85E- 0	CYP19A1, cytochrome p450, family 19, subfamily a, polypeptide 1
15	rs2899472	49303347	A	DOMDEV	0.1614	6.425	4.15E-09	CYP19A1, cytochrome p450, family 19, subfamily a, polypeptide 1
21	rs1022442	21277717	A	GENO_2DF	NA	30.21	2.75E-07	NCAM2, neural cell adhesion molecule 2
10	rs2493624	19102262	G	GENO_2DF	NA	28.61	6.14E-07	
10	rs11015839	19042893	A	GENO_2DF	NA	28.51	6.45E-07	ARL5B, adp-ribosylation factor-like 5b
10	rs11015860	19045045	G	GENO_2DF	NA	28.51	6.45E-07	
1	rs10911736	183689227	A	GENO_2DF	NA	28.16	7.69E-07	
10	rs11015839	19042893	A	ADD	-0.1151	-5.257	7.87E-07	
10	rs11015860	19045045	G	ADD	-0.1151	-5.257	7.87E-07	
10	rs11015939	19057948	A	ADD	-0.1243	-5.219	9.25E-07	
10	rs2495832	19114467	G	ADD	-0.1243	-5.219	9.25E-07	
6	rs12203791	67778209	T	GENO_2DF	NA	27.68	9.76E-07	
10	rs10763625	19074957	T	ADD	-0.124	-5.206	9.78E-07	

**Normal: T-tau**								

**Chromosome**	**SNP**	**Position**	**A1**	**TEST**	**BETA**	**STAT**	**P-value**	**Gene Symbol, Gene Name**

12	rs1997111	117872301	T	GENO_2DF	NA	30.74	2.11E-07	
2	rs17189298	119561787	A	GENO_2DF	NA	27.92	8.66E-07	
18	rs1021599	28469054	T	GENO_2DF	NA	27.88	8.81E-07	

**Normal: P-tau**_**181p**_								

**Chromosome**	**SNP**	**Position**	**A1**	**TEST**	**BETA**	**STAT**	**P-value**	**Gene Symbol, Gene Name**

18	rs1943816	69209602	C	GENO_2DF	NA	31.43	1.50E-07	
2	rs2074955	158686040	C	GENO_2DF	NA	30.78	2.07E-07	UPP2, uridine phosphorylase 2

**MCI: Aβ**_**1-42**_								

**Chromosome**	**SNP**	**Position**	**A1**	**TEST**	**BETA**	**STAT**	**P-value**	**Gene Symbol, Gene Name**

10	rs2986971	30136634	G	GENO_2DF	NA	31.88	1.20E-07	
10	rs2986971	30136634	G	ADD	0.08328	5.228	8.88E-07	

**MCI: T-tau**								

**Chromosome**	**SNP**	**Position**	**A1**	**TEST**	**BETA**	**STAT**	**P-value**	**Gene Symbol, Gene Name**

4	rs2768975	48724658	G	GENO_2DF	NA	30.89	1.96E-07	FLJ21511, hypothetical protein flj21511
4	rs6850199	48695607	A	GENO_2DF	NA	29.93	3.18E-07	FLJ21511, hypothetical protein flj21511
18	rs1445093	48024821	C	GENO_2DF	NA	28.14	7.75E-07	

**MCI: P-tau**_**181p**_								

**Chromosome**	**SNP**	**Position**	**A1**	**TEST**	**BETA**	**STAT**	**P-value**	**Gene Symbol, Gene Name**

7	rs121724	29207970	G	GENO_2DF	NA	31.49	1.45E-07	CHN2, chimerin (chimaerin) 2
8	rs7842088	17629409	G	GENO_2DF	NA	30.73	2.12E-07	MTUS1, mitochondrial tumor suppressor 1

Abbreviations: SNP, single-nucleotide polymorphism; ADD, additive effects of allele dosage; DOMDEV, dominance deviation from additivity, rather specifying that a particular allele is dominant or recessive; GENO_2DF, a 2 df joint test of both additive and dominance; A1, tested allele (minor allele); BETA, regression coefficient; STAT, coefficient t-statistic

### Association of *APOE *with CSF biomarkers

**Figure 6:**
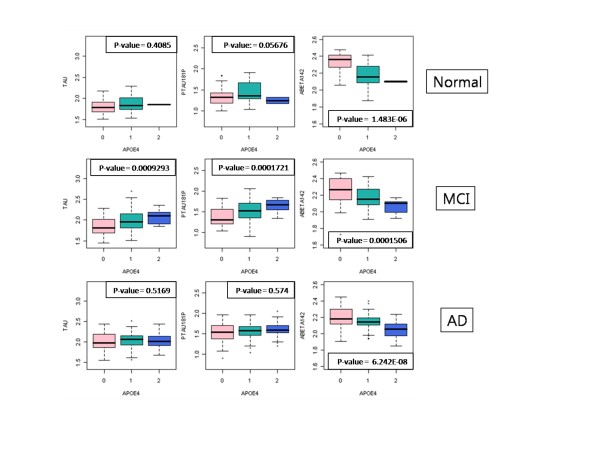
**Boxplots of the *APOE *ε4 copy number with CSF biomarkers in normal, MCI and AD subjects**. The x-axis indicates number of *APOE *ε4 alleles and y-axis indicates CSF biomarkers. P-values are produced by the Kruskal-Wallis test.

**Figure 7:**
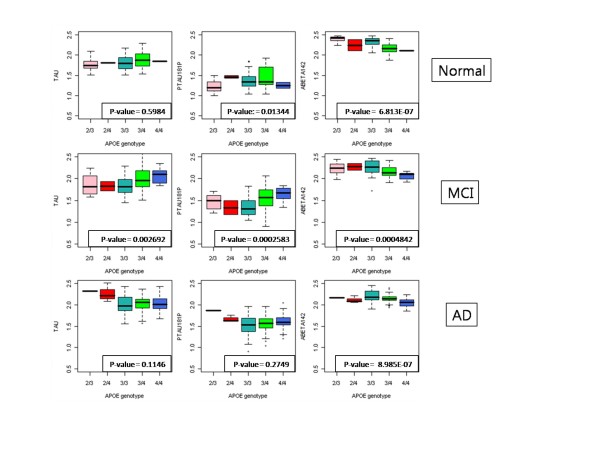
**Boxplots of the *APOE *genotype with CSF biomarkers in normal, MCI and AD subjects**. The x-axis indicates five different *APOE *genotypes and y-axis indicates CSF biomarkers. P-values are produced by the Kruskal-Wallis test.

Previous studies suggesting that CSF Aβ_1-42 _and T-tau levels are correlated with the number of *APOE *ε4 alleles [37]. We analyzed the distribution of levels stratified by diagnosis and the number of *APOE *ε4 in the ADNI cohort after QC (Figure 6) and reached the same conclusion. In the AD group, Aβ_1-42 _level was inversely correlated with *APOE *ε4 allele dose. The *APOE *e4 was not associated with T-tau or P-tau_181P _levels. Analysis of *APOE *genotypes showed that ε4/ε4 is associated with CSF biomarker level of Aβ_1-42 _, T-tau and P-tau_181P _(Figure 7).

### Pathway analysis of CSF biomarkers

We ran ALIGATOR to identify top gene ontology terms associated with genes containing SNPs with higher statistical significance, and summarized the results in Additional file 7. We found cerebral cortex development, methionine metabolic process, actinin binding, and pallium development to be among the most significant gene ontology terms associated with CSF biomarker level of Aβ_1-42 _in normal subjects. Elevation in Aβ in the cerebral cortex has been implicated in the pathophysiology of AD but its mechanism of action is unknown [38]. It has been known that mammals have a fully developed cortex, but the structure it evolved from pallium which is present in all vertebrates as well as the most primitive ones [39]. The medial pallium forms the precursor of the hippocampus. Since hippocampal disruption is one of the earliest signs for AD, pallium development might be involved in the pathophysiology AD.

### E-SNP analysis of CSF biomarkers

We collected eSNPs (SNPs known to be associated with the expression level of some genes) in several published expression quantitative linkage (eQTL) studies, and examined regions around significant SNPs (p < 10^-4^) (Additional file 8). We found significant SNPs from Dixon *et al*. [29], Stranger *et al*. [31], and Gibbs *et al*. [33], but we could not find any top SNPs that are associated with gene expression from other papers [30,32]. Dixon *et al*. [29] used lymphoblastoid cell lines (LCLs) derived from children both with and without asthma.

### NCAM2 (Neural Cell Adhesion Molecule 2)

We obtained strong evidence that NCAM2 genotypes are associated with Aβ_1-42 _levels. *NCAM2 *(Ensemble: ENSG00000154654, OMIM: 602040;) is a 541Kb gene at 21q21.1 with no known alternative spicing forms. Novartis SymAtlas human tissue survey shows the gene (Affymetrix probeset ID 205669_at) is ubiquitously expressed and is highly expressed in cardiac myocytes, blood cells, and appendix. Among neuronal tissues *NCAM2 *has higher expression levels in prefrontal cortex, superior cervical ganglion, and hypothalamus. The transcript (Ensembl: ENST00000400546) consists of 18 exons and encodes a 93 k-Da, 835-residue plasma membrane protein (NP_004531.2). The *NCAM2 *protein architecture includes 5 IgC2 (Immunoglobulin C-2 type) domains followed by two FN3 (fibronectin type 3) domains and a transmembrane domain. The gene is conserved in chimpanzee, dog, cow, mouse, rat, chicken, zebrafish, and fruit fly; the eight-domain protein architecture is also conserved in all these organisms except for cow which has only four IgC2 domains. Little is known about *NCAM2 *except that the protein interacts with prion protein [40] and estrogen receptor 1 (ESR1) [41], is involved in neuron adhesion and fasciculation of neurons, and may be involved in AD [36], prion disease, and Down syndrome [42,43].

### TOMM40 (translocase of outer mitochondrial membrane 40 homolog (yeast)) and CYP19A1 (Cytochrome P450, family 19, subfamily A, polypeptide 1)

It is interesting to note that we found no significant association in *APOE *(rs769451, chr19: 50102751, p = 0.6682 for association with Aβ_1-42 _level in normal subjects), but a SNP with strong association in the nearby *TOMM40 *(intronic SNP rs2075650, chr19:50087459, p = 3.03 × 10^-7 ^for association with Aβ_1-42 _level in normal subjects), when age and the number of APOE e4 alleles were not included in the regression. The *TOMM40 *gene is related to how easily molecules can get into and out of the surface of the mitochondria, the energy center of cells. This gene is a transporter of proteins across the mitochondrial membrane, and Sortillin-related receptor, which functions to partition amyloid precursor protein away from β-secretase and -secretase [44]. This is consistent with observations that levels are reduced in the brains of patients with Alzheimer's disease and MCI [44-46]. The *TOMM40 *gene has been reported in numerous studies in the study of AD genetics; for example, Yu *et al*. [47] reported possibility that loci in the *TOMM40 *gene may have a less effect on the risk for LOAD in Caucasians [47], and recently Roses *et al*. [48] found evidence supporting a poly-T polymorphism (rs10524523, chr19:50094889) in *TOMM40 *affecting the AD age of onset in two independent clinical cohorts. The potential association of *TOMM40 *and Aβ_1-42 _may be how the gene affects the risk and onset age of AD and should be further investigated.

The *CYP19A1 *gene is localized on chromosome 15q21.2 and spans 123 kb. This gene encodes a member of the cytochrome P450 superfamily of enzymes. Cytochrome P450 aromatase is an enzyme that catalyses the conversion of androgens, such as testosterone, to oestrogens, which act as sex steroid hormones but also function during growth and differentiation. There are high levels of expression in both the gonads and the brain [35]. Huang *et al*. [35] indicated an increased risk associated with SNP rs2899472 in the total number of AD patients, which was amplified in APOE ε4 carriers.

## Conclusions

Our analysis of the ADNI genome-wide association study identified several putative loci that are in genetic association with Aβ_1-42_, T-tau and P-tau_181P _levels in cerebrospinal fluids. In particular an intronic SNP rs1022442 of gene *NCAM2 *is close to genome-wide significance in association with Aβ_1-42 _in normal subjects. Although the gene is poorly characterized in the literature, prior studies have implicated roles of *NCAM2 *in prion disease, Down syndrome, and AD. Our findings suggest *NCAM2 *could be part of the pathway on the pathogenesis of senile plaques in human brains with AD.

With only 119 normal subjects and 410 overall, the GWAS dataset is clearly underpowered. The most significant associations were identified using normal subjects since the variances of the CSF biomarker levels are much smaller in MCI and AD subjects due to dementia. Nonetheless, increasing the number of CSF biomarker measurements is challenging especially for normal subjects. An alternative that will substantially increase the sample size is to examine protein levels in blood instead of CSF, given levels of these proteins in blood are informative about AD pathology or prognosis [49].

Our analysis clearly demonstrates that quantitative trait linkage of biomarkers via genome-wide screening can reveal additional insights into the mechanism that connects known genetic factors to the disease [50]. Moving along this path requires further efforts by the research community towards larger sample size and accrual of additional biomarkers in AD and other neurodegenerative disorders.

## Competing interests

The authors declare that they have no competing interests.

## Authors' contributions

M-RH analyzed and interpreted the data, and wrote the manuscript. L-SW conceived the study, analyzed and interpreted the data, and drafted the manuscript. GDS assisted in data analysis and interpretation, and made critical revisions to the manuscript. All authors read and approved the final manuscript.

## Pre-publication history

The pre-publication history for this paper can be accessed here:

http://www.biomedcentral.com/1471-2377/10/90/prepub

## Supplementary Material

Additional file 1 **Demographic, clinical and biomarker data for each subject group before removing 20 outliers (n = 410: Normal, MCI and AD)**. Click here for file

Additional file 2 **Ethnic and Racial summary for ADNI data**. Click here for file

Additional file 3 **Population stratification assessments using PCA. Colors indicate different ethnic (A) and racial (B) groups**. The x-axis indicates the first principal component (PC0) and y-axis indicates the second principal component (PC1). Click here for file

Additional file 4 **Log10(CSF levels) for each subject group after removing 20 outliers (n = 390: Normal, MCI and AD)**. Click here for file

Additional file 5 **Log10(CSF levels) for each subject group before removing 20 outliers (n = 410: Normal, MCI and AD)**. Click here for file

Additional file 6 **Quantile-Quantile plots of three CSF biomarkers in case/control (left: GENO_2DF test, middle: DOMDEV test, right: ADD test)**. The x-axis indicates quantile values (-log10(quantile values)) and y-axis indicates p-values (-log10(observed p-values)). Click here for file

Additional file 7 **Enriched GO Categories (Top 10 GO categories with p-value < 10^-2^)**. Click here for file

Additional file 8 **Significant SNPs (p-value < 10^-4^) known to be associated with gene expression in published eQTL studies**. Click here for file
