# Supplementary material for: Genome-wide association reveals genetic effects on human Aβ42 and τ protein levels in cerebrospinal fluids: a case control study
Source: BMC Neurol. 2010 Oct 8;10:90. doi: 10.1186/1471-2377-10-90 (PMC2964649; doi:10.1186/1471-2377-10-90)
Supplement: Additional file 1 — Demographic, clinical and biomarker data for each subject group before removing 20 outliers (n = 410: Normal, MCI and AD). [file 1471-2377-10-90-S1.DOC]

**Additional file 1. Demographic, clinical and biomarker data for each subject group before removing 20 outliers (n = 410: Normal, MCI and AD)**

| **Normal (n = 119)** | **Mean** |
| --- | --- |
| Age (year) | 71.4 |
| CSF Aβ_1-42_ levels (pg/ml) | 205.8 |
| CSF P-tau_181P_ levels (pg/ml) | 24.8 |
| CSF T-tau levels (pg/ml) | 70.3 |
| Last MMSE | 29 |
|  | **N** |
| Male | 63 |
| Female | 56 |
| **MCI (n = 115)** | **Mean** |
| Age (year) | 70.3 |
| CSF Aβ_1-42_ levels (pg/ml) | 173.3 |
| CSF P-tau_181P_ levels (pg/ml) | 33.3 |
| CSF T-tau levels (pg/ml) | 98.2 |
| Last MMSE | 26.5 |
|  | **N** |
| Male | 77 |
| Female | 38 |
| **AD (n = 176)** | **Mean** |
| Age (year) | 71 |
| CSF Aβ_1-42_ levels (pg/ml) | 144.4 |
| CSF P-tau_181P_ levels (pg/ml) | 40.9 |
| CSF T-tau levels (pg/ml) | 117.5 |
| Last MMSE | 21.1 |
|  | **N** |
| Male | 107 |
| Female | 69 |
| Abbreviations: CSF, cerebral spinal fluid; MMSE, Mini Mental State Examination | |
