# Supplementary material for: Genome-wide association reveals genetic effects on human Aβ42 and τ protein levels in cerebrospinal fluids: a case control study
Source: BMC Neurol. 2010 Oct 8;10:90. doi: 10.1186/1471-2377-10-90 (PMC2964649; doi:10.1186/1471-2377-10-90)
Supplement: Additional file 2 — Ethnic and Racial summary for ADNI data. [file 1471-2377-10-90-S2.DOC]

**Additional file 2. Ethnic and Racial summary for ADNI data**

| **Clinical data** | **ADNI** |
| --- | --- |
| **Sample** | 818 |
|  | |
| **Ethnic Category** | |
| Hispanic or Latino | 19 |
| Not Hispanic or Latino | 790 |
| Unknown | 9 |
|  | |
| **Racial Categories** | |
| American Indian or Alaskan Native | 1 |
| Asian | 14 |
| Native Hawaiian or Other Pacific Islander | 0 |
| Black or African American | 39 |
| White | 761 |
| More than one race | 3 |
| Unknown | 0 |
| Abbreviations: ADNI, Alzheimer’s disease Neuroimaging Initiative | |
