# Supplementary material for: Genome-wide association reveals genetic effects on human Aβ42 and τ protein levels in cerebrospinal fluids: a case control study
Source: BMC Neurol. 2010 Oct 8;10:90. doi: 10.1186/1471-2377-10-90 (PMC2964649; doi:10.1186/1471-2377-10-90)

**Additional file 3. Population stratification assessments using PCA. Colors indicate different ethnic (A) and racial (B) groups. The x-axis indicates the first principal component (PC0) and y-axis indicates the second principal component (PC1).**


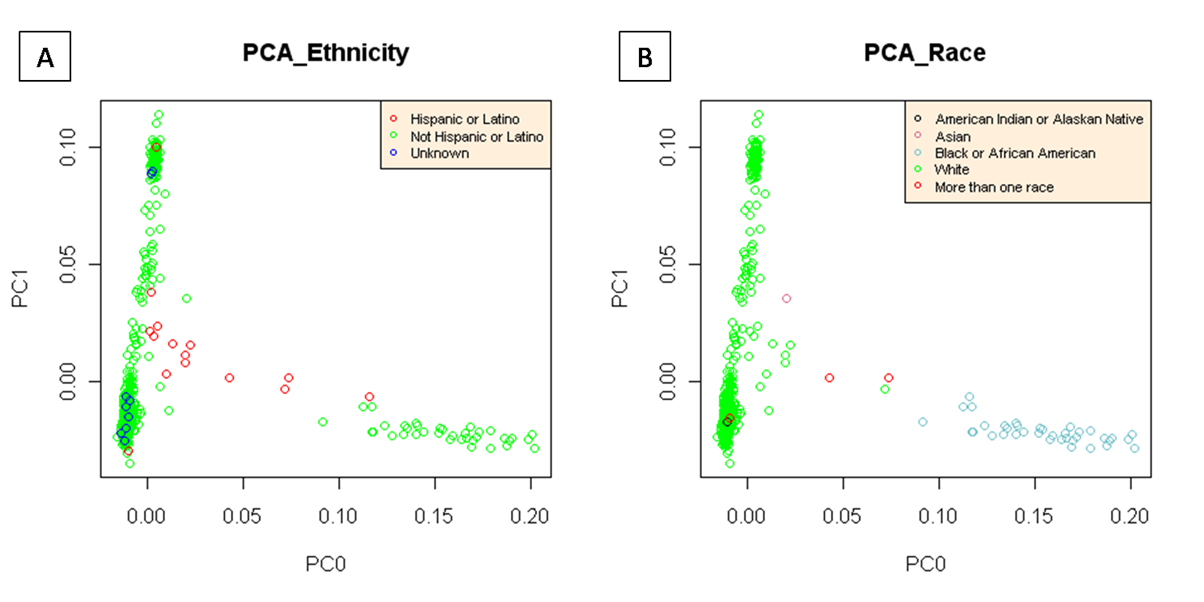

Supplement: Additional file 3 — Population stratification assessments using PCA. Colors indicate different ethnic (A) and racial (B) groups. The x-axis indicates the first principal component (PC0) and y-axis indicates the second principal component (PC1). [file 1471-2377-10-90-S3.DOC]
