# Supplementary material for: Genome-wide association reveals genetic effects on human Aβ42 and τ protein levels in cerebrospinal fluids: a case control study
Source: BMC Neurol. 2010 Oct 8;10:90. doi: 10.1186/1471-2377-10-90 (PMC2964649; doi:10.1186/1471-2377-10-90)
Supplement: Additional file 4 — Log10(CSF levels) for each subject group after removing 20 outliers (n = 390: Normal, MCI and AD). [file 1471-2377-10-90-S4.DOC]

**Additional file 4. Log10(CSF levels) for each subject group after removing 20 outliers (n = 390: Normal, MCI and AD)**

|  | | | | | |  |
| --- | --- | --- | --- | --- | --- | --- |
| **Normal (n = 109)** | **Mean** | | **SD** |  |  | |
| log10(CSF) Aβ_1-42_ levels (pg/ml) | 2.3 | | 0.1 |  |  | |
| log10(CSF) P-tau_181P_ levels (pg/ml) | 1.4 | | 0.2 |  |  | |
| log10(CSF) T-tau levels (pg/ml) | 1.8 | | 0.2 |  |  | |
| **MCI (n = 109)** | **Mean** | | **SD** |  |  | |
| log10(CSF) Aβ_1-42_ levels (pg/ml) | 2.2 | | 0.2 |  |  | |
| log10(CSF) P-tau_181P_ levels (pg/ml) | 1.5 | | 0.2 |  |  | |
| log10(CSF) T-tau levels (pg/ml) | 1.9 | | 0.2 |  |  | |
| **AD (n = 172)** | **Mean** | | **SD** |  |  | |
| log10(CSF) Aβ_1-42_ levels (pg/ml) | 2.1 | | 0.1 |  |  | |
| log10(CSF) P-tau_181P_ levels (pg/ml) | 1.6 | | 0.2 |  |  | |
| log10(CSF) T-tau levels (pg/ml) | 2.0 | | 0.2 |  |  | |
| Abbreviations: CSF, cerebral spinal fluid |  |  |  |  |  | |
