# Supplementary material for: Genome-wide association reveals genetic effects on human Aβ42 and τ protein levels in cerebrospinal fluids: a case control study
Source: BMC Neurol. 2010 Oct 8;10:90. doi: 10.1186/1471-2377-10-90 (PMC2964649; doi:10.1186/1471-2377-10-90)

**Additional file 6. Quantile-Quantile plots of three CSF biomarkers in case/control.**

(left: GENO_2DF test, middle: DOMDEV test, right: ADD test). The x-axis indicates quantile values (–log10(quantile values)) and y-axis indicates p-values (–log10(observed p-values))


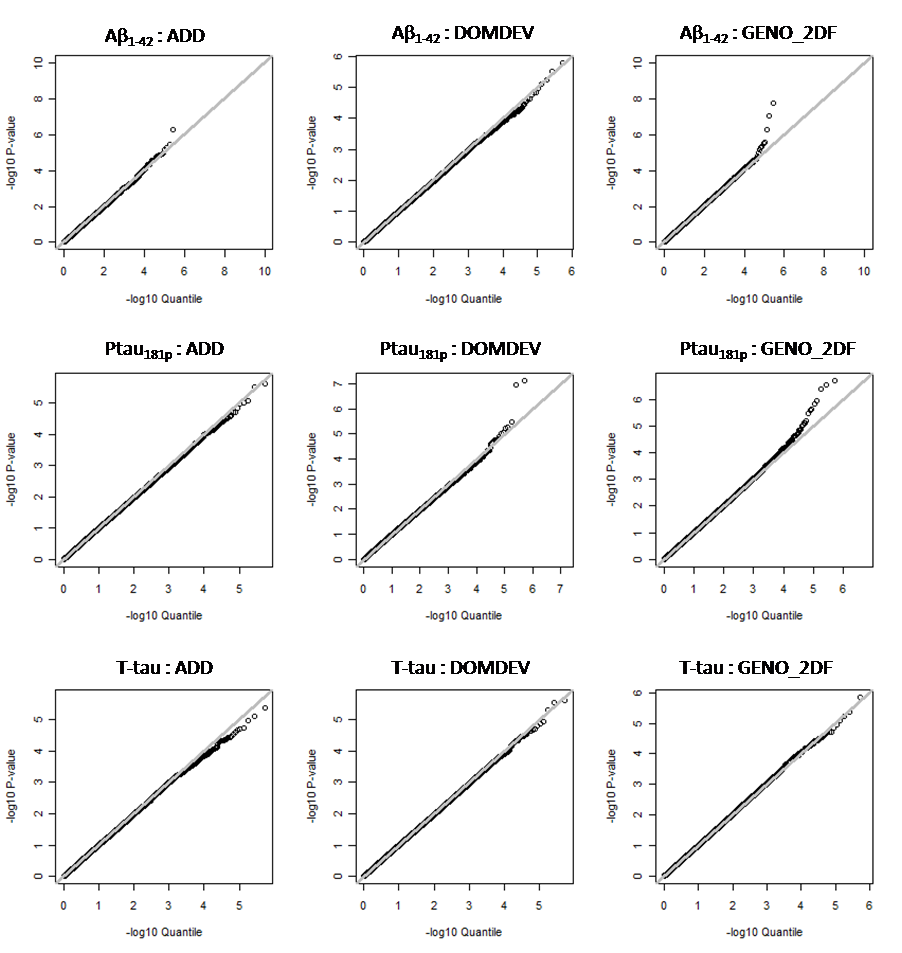

Supplement: Additional file 6 — Quantile-Quantile plots of three CSF biomarkers in case/control (left: GENO_2DF test, middle: DOMDEV test, right: ADD test). The x-axis indicates quantile values (-log10(quantile values)) and y-axis indicates p-values (-log10(observed p-values)). [file 1471-2377-10-90-S6.DOC]
