# Supplementary material for: Genome-wide association reveals genetic effects on human Aβ42 and τ protein levels in cerebrospinal fluids: a case control study
Source: BMC Neurol. 2010 Oct 8;10:90. doi: 10.1186/1471-2377-10-90 (PMC2964649; doi:10.1186/1471-2377-10-90)
Supplement: Additional file 7 — Enriched GO Categories (Top 10 GO categories with p-value < 10-2). [file 1471-2377-10-90-S7.DOC]

**Additional file 7. Enriched GO Categories (Top 10 GO categories with p-value < 10^-2^).**

| **Normal: T-tau** | | | | | | | |
| --- | --- | --- | --- | --- | --- | --- | --- |
| **GO Category^a^** | **Type^b^** | **Total Genes in Category** | **No. Of Genes on List** | **Expected No. of Genes on List** | **P-value^c^** | **Expected Hits per Study^d^** | **Function** |
|
| GO:0004064 | FUNCTION | 3 | 2 | 0.02 | 0 | 0.52 | arylesterase activity |
| GO:0048568 | PROCESS | 28 | 3 | 0.35 | 0.0024 | 3.66 | embryonic organ development |
| GO:0035108 | PROCESS | 48 | 4 | 0.64 | 0.0028 | 4.17 | limb morphogenesis |
| GO:0035107 | PROCESS | 48 | 4 | 0.64 | 0.0028 | 4.17 | appendage morphogenesis |
| GO:0060173 | PROCESS | 50 | 4 | 0.65 | 0.0036 | 5.2 | limb development |
| GO:0048736 | PROCESS | 50 | 4 | 0.65 | 0.0036 | 5.2 | appendage development |
| GO:0048048 | PROCESS | 8 | 2 | 0.1 | 0.0046 | 6.45 | embryonic eye morphogenesis |
| GO:0006897 | PROCESS | 160 | 6 | 1.74 | 0.0052 | 7.16 | endocytosis |
| GO:0010324 | PROCESS | 160 | 6 | 1.74 | 0.0052 | 7.16 | membrane invagination |
| GO:0031901 | CELLULAR | 26 | 2 | 0.11 | 0.0062 | 8.42 | early endosome membrane |
| **Normal: P-tau_181P_** | | | | | | | |
| **GO Category^a^** | **Type^b^** | **Total Genes in Category** | **No. Of Genes on List** | **Expected No. of Genes on List** | **P-value^c^** | **Expected Hits per Study^d^** | **Function** |
|
| GO:0006508 | PROCESS | 835 | 15 | 6.26 | 0.0008 | 1.82 | proteolysis |
| GO:0048566 | PROCESS | 8 | 2 | 0.16 | 0.0068 | 9.94 | embryonic gut development |
| GO:0008233 | FUNCTION | 472 | 10 | 4.14 | 0.0086 | 12.43 | peptidase activity |
| GO:0004175 | FUNCTION | 307 | 7 | 2.38 | 0.0088 | 12.7 | endopeptidase activity |
| **Normal: Aβ_1-42_** | | | | | | | |
| **GO Category^a^** | **Type^b^** | **Total Genes in Category** | **No. Of Genes on List** | **Expected No. of Genes on List** | **P-value^c^** | **Expected Hits per Study^d^** | **Function** |
|
| GO:0021987 | PROCESS | 15 | 2 | 0.18 | 8.00E-03 | 11.81 | cerebral cortex development |
| GO:0006555 | PROCESS | 11 | 2 | 0.17 | 0.0082 | 12.06 | methionine metabolic process |
| GO:0042805 | FUNCTION | 4 | 2 | 0.17 | 0.0092 | 13.35 | actinin binding |
| GO:0021543 | PROCESS | 20 | 2 | 0.19 | 0.0096 | 13.91 | pallium development |
| **MCI: T-tau** | | | | | | | |
| **GO Category^a^** | **Type^b^** | **Total Genes in Category** | **No. Of Genes on List** | **Expected No. of Genes on List** | **P-value^c^** | **Expected Hits per Study^d^** | **Function** |
|
| GO:0006506 | PROCESS | 26 | 2 | 0.09 | 0.0028 | 4.58 | GPI anchor biosynthetic process |
| GO:0006505 | PROCESS | 26 | 2 | 0.09 | 0.0028 | 4.58 | GPI anchor metabolic process |
| GO:0046489 | PROCESS | 32 | 2 | 0.11 | 0.0052 | 7.78 | phosphoinositide biosynthetic process |
| GO:0007528 | PROCESS | 9 | 2 | 0.16 | 0.0078 | 11.09 | neuromuscular junction development |
| GO:0007090 | PROCESS | 9 | 2 | 0.17 | 0.0082 | 11.62 | regulation of S phase of mitotic cell cycle |
| GO:0006029 | PROCESS | 31 | 3 | 0.53 | 0.0096 | 13.51 | proteoglycan metabolic process |
| **MCI: P-tau_181P_** | | | | | | | |
| **GO Category^a^** | **Type^b^** | **Total Genes in Category** | **No. Of Genes on List** | **Expected No. of Genes on List** | **P-value^c^** | **Expected Hits per Study^d^** | **Function** |
|
| GO:0051347 | PROCESS | 140 | 5 | 0.88 | 0.0018 | 2.68 | positive regulation of transferase activity |
| GO:0010906 | PROCESS | 15 | 2 | 0.1 | 0.0024 | 3.42 | regulation of glucose metabolic process |
| GO:0001518 | CELLULAR | 12 | 2 | 0.08 | 0.0024 | 3.42 | voltage-gated sodium channel complex |
| GO:0034706 | CELLULAR | 12 | 2 | 0.08 | 0.0024 | 3.42 | sodium channel complex |
| GO:0015980 | PROCESS | 74 | 3 | 0.29 | 0.0032 | 4.36 | energy derivation by oxidation of organic compounds |
| GO:0005248 | FUNCTION | 15 | 2 | 0.09 | 0.0032 | 4.36 | voltage-gated sodium channel activity |
| GO:0046320 | PROCESS | 15 | 2 | 0.1 | 0.004 | 5.26 | regulation of fatty acid oxidation |
| GO:0045444 | PROCESS | 22 | 2 | 0.09 | 0.0044 | 5.67 | fat cell differentiation |
| GO:0004860 | FUNCTION | 26 | 2 | 0.11 | 0.0044 | 5.67 | protein kinase inhibitor activity |
| GO:0019210 | FUNCTION | 27 | 2 | 0.12 | 0.0046 | 5.91 | kinase inhibitor activity |
| **MCI: Aβ_1-42_** | | | | | | | |
| **GO Category^a^** | **Type^b^** | **Total Genes in Category** | **No. Of Genes on List** | **Expected No. of Genes on List** | **P-value^c^** | **Expected Hits per Study^d^** | **Function** |
|
| GO:0006633 | PROCESS | 64 | 7 | 1.26 | 0 | 0.54 | fatty acid biosynthetic process |
| GO:0042832 | PROCESS | 3 | 2 | 0.03 | 0.0006 | 1.66 | defense response to protozoan |
| GO:0001562 | PROCESS | 3 | 2 | 0.03 | 0.0006 | 1.66 | response to protozoan |
| GO:0006631 | PROCESS | 154 | 10 | 2.7 | 0.0008 | 2.05 | fatty acid metabolic process |
| GO:0046394 | PROCESS | 75 | 7 | 1.49 | 0.0008 | 2.05 | carboxylic acid biosynthetic process |
| GO:0016053 | PROCESS | 75 | 7 | 1.49 | 0.0008 | 2.05 | organic acid biosynthetic process |
| GO:0030856 | PROCESS | 7 | 2 | 0.06 | 0.0008 | 2.05 | regulation of epithelial cell differentiation |
| GO:0002313 | PROCESS | 4 | 2 | 0.14 | 0.0014 | 3.2 | mature B cell differentiation during immune response |
| GO:0002312 | PROCESS | 4 | 2 | 0.14 | 0.0014 | 3.2 | B cell activation during immune response |
| GO:0002335 | PROCESS | 4 | 2 | 0.14 | 0.0014 | 3.2 | mature B cell differentiation |
| **AD: T-tau** | | | | | | | |
| **GO Category^a^** | **Type^b^** | **Total Genes in Category** | **No. Of Genes on List** | **Expected No. of Genes on List** | **P-value^c^** | **Expected Hits per Study^d^** | **Function** |
|
| GO:0019882 | PROCESS | 43 | 3 | 0.12 | 0 | 0.52 | antigen processing and presentation |
| GO:0048002 | PROCESS | 19 | 2 | 0.06 | 0.0014 | 2.51 | antigen processing and presentation of peptide antigen |
| GO:0016853 | FUNCTION | 99 | 3 | 0.4 | 0.0056 | 7.8 | isomerase activity |
| GO:0015833 | PROCESS | 27 | 2 | 0.14 | 0.007 | 9.5 | peptide transport |
| **AD: P-tau_181P_** | | | | | | | |
| **GO Category^a^** | **Type^b^** | **Total Genes in Category** | **No. Of Genes on List** | **Expected No. of Genes on List** | **P-value^c^** | **Expected Hits per Study^d^** | **Function** |
|
| GO:0009628 | PROCESS | 187 | 8 | 1.98 | 0.0002 | 0.87 | response to abiotic stimulus |
| GO:0016742 | FUNCTION | 6 | 2 | 0.09 | 0.002 | 3.78 | hydroxymethyl-, formyl- and related transferase activity |
| GO:0004372 | FUNCTION | 7 | 2 | 0.09 | 0.002 | 3.78 | glycine hydroxymethyltransferase activity |
| GO:0048266 | PROCESS | 4 | 2 | 0.13 | 0.002 | 3.78 | behavioral response to pain |
| GO:0040008 | PROCESS | 198 | 7 | 1.87 | 0.0026 | 4.71 | regulation of growth |
| GO:0015893 | PROCESS | 11 | 2 | 0.06 | 0.0026 | 4.71 | drug transport |
| GO:0046112 | PROCESS | 10 | 2 | 0.19 | 0.004 | 6.88 | nucleobase biosynthetic process |
| GO:0030261 | PROCESS | 19 | 2 | 0.11 | 0.0042 | 7.17 | chromosome condensation |
| GO:0034404 | PROCESS | 12 | 2 | 0.19 | 0.0046 | 7.74 | nucleobase, nucleoside and nucleotide biosynthetic process |
| GO:0034654 | PROCESS | 12 | 2 | 0.19 | 0.0046 | 7.74 | nucleobase, nucleoside |
| **AD: Aβ_1-42_** | | | | | | | |
| **GO Category^a^** | **Type^b^** | **Total Genes in Category** | **No. Of Genes on List** | **Expected No. of Genes on List** | **P-value^c^** | **Expected Hits per Study^d^** | **Function** |
|
| GO:0034961 | PROCESS | 1648 | 20 | 11.3 | 7.40E-03 | 11.47 | cellular biopolymer biosynthetic process |
| GO:0043284 | PROCESS | 1648 | 20 | 11.3 | 0.0074 | 11.47 | biopolymer biosynthetic process |
| GO:0051252 | PROCESS | 1816 | 23 | 13.74 | 0.009 | 13.6 | regulation of RNA metabolic process |

^a^ List of significantly overrepresented GO categories for three CSF biomarkers in case/control (cutoff for significant SNPs: p-value < 10-3).

^b^ The type of category.

^c^ The category-specific p-value for over-representation.

^d^ The expected number of categories with a category-specific overrepresentation p-value at least as significant as that observed in the absence of any true overrepresentation.
