# Supplementary material for: Genome-wide association reveals genetic effects on human Aβ42 and τ protein levels in cerebrospinal fluids: a case control study
Source: BMC Neurol. 2010 Oct 8;10:90. doi: 10.1186/1471-2377-10-90 (PMC2964649; doi:10.1186/1471-2377-10-90)
Supplement: Additional file 8 — Significant SNPs (p-value < 10-4) known to be associated with gene expression in published eQTL studies. [file 1471-2377-10-90-S8.DOC]

**Additional file 8. Significant SNPs (p-value < 10^-4^) known to be associated with gene expression in published eQTL studies.**

|  |  | |  |  |  |  |  |  |  |  |  |  |  |  |
| --- | --- | --- | --- | --- | --- | --- | --- | --- | --- | --- | --- | --- | --- | --- |
| **Normal: Aβ_1-42_** | | |  |  |  |  |  |  |  |  |  |  |  |  |
| **CHR** | **SNP** | | **POSITION** | **A1** | **TEST** | **P-value** | **LCL^a^** | **EBV.transformed.LCL_Cis_JPT ^b^** | **EBV.transformed.LCL_Cis_CHB.JPT ^b^** | **EBV.transformed.LCL_Cis_CEU.CHB.JPT.YRI ^b^** | **HumanBrainTissue_pons ^c^** | **HumanBrainTissue_crblm ^c^** | **HumanBrainTissue_fctx ^c^** | **HumanBrainTissue_tctx ^c^** |
| 10 | rs7905063 | | 52634596 | C | DOMDEV | 1.72E-05 |  |  |  |  |  |  |  |  |
| 10 | rs7905063 | | 52634596 | C | GENO_2DF | 3.87E-05 |  |  |  |  |  |  |  |  |
| 6 | rs9373409 | | 144328911 | T | DOMDEV | 1.60E-05 |  |  |  |  |  |  |  |  |
| 6 | rs9373409 | | 144328911 | T | GENO_2DF | 3.54E-05 |  |  |  |  |  |  |  |  |
| **Normal: T-tau** | | |  |  |  |  |  |  |  |  |  |  |  |  |
| **CHR** | **SNP** | | **POSITION** | **A1** | **TEST** | **P-value** | **LCL^a^** | **EBV.transformed.LCL_Cis_JPT ^b^** | **EBV.transformed.LCL_Cis_CHB.JPT ^b^** | **EBV.transformed.LCL_Cis_CEU.CHB.JPT.YRI ^b^** | **HumanBrainTissue_pons ^c^** | **HumanBrainTissue_crblm ^c^** | **HumanBrainTissue_fctx ^c^** | **HumanBrainTissue_tctx ^c^** |
| 17 | rs2466529 | | 68434131 | A | DOMDEV | 2.89E-05 |  |  |  |  |  |  |  |  |
| 17 | rs2466529 | | 68434131 | A | GENO_2DF | 4.96E-05 |  |  |  |  |  |  |  |  |
| 2 | rs17020183 | | 37159655 | A | GENO_2DF | 1.00E-05 |  |  |  |  |  |  |  |  |
| 2 | rs7349378 | | 174481174 | T | ADD | 8.13E-05 |  |  |  |  |  |  |  |  |
| 3 | rs1702122 | | 129566022 | C | ADD | 3.93E-05 |  |  |  |  |  |  |  |  |
| 3 | rs1702122 | | 129566022 | C | GENO_2DF | 8.54E-05 |  |  |  |  |  |  |  |  |
| **Normal: P-tau_181P_** | | |  |  |  |  |  |  |  |  |  |  |  |  |
| **CHR** | **SNP** | | **POSITION** | **A1** | **TEST** | **P-value** | **LCL^a^** | **EBV.transformed.LCL_Cis_JPT ^b^** | **EBV.transformed.LCL_Cis_CHB.JPT ^b^** | **EBV.transformed.LCL_Cis_CEU.CHB.JPT.YRI ^b^** | **HumanBrainTissue_pons ^c^** | **HumanBrainTissue_crblm ^c^** | **HumanBrainTissue_fctx ^c^** | **HumanBrainTissue_tctx ^c^** |
| 10 | rs4918082 | | 95849919 | G | ADD | 9.20E-05 |  |  |  |  |  |  |  |  |
| 12 | rs2306393 | | 66995028 | C | ADD | 7.31E-05 |  |  |  |  |  |  |  |  |
| 12 | rs962976 | | 67006894 | G | ADD | 7.31E-05 |  |  |  |  |  |  |  |  |
| 19 | rs9304718 | | 56703742 | C | GENO_2DF | 5.11E-05 |  |  |  |  | SIGLEC6 | SIGLEC6 |  |  |
| 1 | rs3753519 | | 207942138 | A | ADD | 9.73E-05 |  |  |  |  |  |  |  |  |
| 6 | rs346300 | | 80630745 | T | ADD | 8.05E-05 |  |  |  |  |  |  |  |  |
| 6 | rs346300 | | 80630745 | T | GENO_2DF | 9.21E-05 |  |  |  |  |  |  |  |  |
| **MCI: Aβ_1-42_** | | |  |  |  |  |  |  |  |  |  |  |  |  |
| **CHR** | **SNP** | | **POSITION** | **A1** | **TEST** | **P-value** | **LCL^a^** | **EBV.transformed.LCL_Cis_JPT ^b^** | **EBV.transformed.LCL_Cis_CHB.JPT ^b^** | **EBV.transformed.LCL_Cis_CEU.CHB.JPT.YRI ^b^** | **HumanBrainTissue_pons ^c^** | **HumanBrainTissue_crblm ^c^** | **HumanBrainTissue_fctx ^c^** | **HumanBrainTissue_tctx ^c^** |
| 10 | rs10510060 | | 121853460 | A | ADD | 4.97E-05 |  |  |  |  |  |  |  |  |
| 11 | rs2276035 | | 119851570 | T | ADD | 7.24E-05 |  |  |  |  |  |  |  |  |
| 11 | rs2276035 | | 119851570 | T | DOMDEV | 6.58E-05 |  |  |  |  |  |  |  |  |
| 11 | rs483176 | | 95261371 | T | ADD | 5.75E-05 |  |  |  |  |  |  |  |  |
| 11 | rs483176 | | 95261371 | T | DOMDEV | 9.05E-06 |  |  |  |  |  |  |  |  |
| 11 | rs483176 | | 95261371 | T | GENO_2DF | 1.48E-05 |  |  |  |  |  |  |  |  |
| 12 | rs11170682 | | 52461288 | C | DOMDEV | 1.61E-05 |  |  |  |  |  |  |  |  |
| 12 | rs11170682 | | 52461288 | C | GENO_2DF | 9.42E-06 |  |  |  |  |  |  |  |  |
| 12 | rs1249963 | | 53276390 | G | ADD | 8.51E-05 |  |  |  |  |  |  |  |  |
| 12 | rs3759217 | | 12759719 | T | ADD | 6.28E-05 |  |  |  |  |  |  |  |  |
| 12 | rs5019656 | | 70608710 | T | ADD | 5.88E-05 |  |  |  |  |  |  |  |  |
| 12 | rs6582065 | | 70579433 | C | ADD | 5.87E-05 |  |  |  |  |  |  |  |  |
| 15 | rs10519246 | | 47995800 | T | ADD | 6.47E-05 |  |  |  |  |  |  |  |  |
| 15 | rs10519246 | | 47995800 | T | DOMDEV | 1.91E-05 |  |  |  |  |  |  |  |  |
| 15 | rs10519246 | | 47995800 | T | GENO_2DF | 3.94E-05 |  |  |  |  |  |  |  |  |
| 17 | rs8077638 | | 1587543 | T | DOMDEV | 8.62E-05 |  |  |  |  |  |  |  |  |
| 1 | rs10489691 | | 231578985 | G | ADD | 1.47E-05 |  |  |  |  |  |  |  |  |
| 1 | rs10489691 | | 231578985 | G | GENO_2DF | 2.23E-05 |  |  |  |  |  |  |  |  |
| 1 | rs12137761 | | 176203148 | G | ADD | 5.35E-05 |  |  |  |  |  |  |  |  |
| 1 | rs3795375 | | 231581557 | T | ADD | 1.35E-05 |  |  |  |  |  |  |  |  |
| 1 | rs3795375 | | 231581557 | T | GENO_2DF | 1.83E-05 |  |  |  |  |  |  |  |  |
| 1 | rs831768 | | 199415656 | A | ADD | 5.80E-05 |  |  |  |  |  |  |  |  |
| 20 | rs17310467 | | 33009277 | G | ADD | 7.41E-05 |  |  |  |  |  |  |  |  |
| 20 | rs867186 | | 33228215 | G | ADD | 6.48E-05 |  |  |  |  |  |  |  |  |
| 2 | rs4671979 | | 54932746 | T | ADD | 3.67E-05 |  |  |  |  |  |  |  |  |
| 2 | rs4671979 | | 54932746 | T | GENO_2DF | 8.28E-05 |  |  |  |  |  |  |  |  |
| 3 | rs11130070 | | 45591919 | T | ADD | 4.07E-05 |  |  |  |  |  |  |  |  |
| 3 | rs11130070 | | 45591919 | T | DOMDEV | 1.28E-05 |  |  |  |  |  |  |  |  |
| 3 | rs11130070 | | 45591919 | T | GENO_2DF | 1.86E-05 |  |  |  |  |  |  |  |  |
| 3 | rs6549882 | | 28744829 | G | ADD | 4.44E-05 |  |  |  |  |  |  |  |  |
| 3 | rs6549882 | | 28744829 | G | GENO_2DF | 4.48E-05 |  |  |  |  |  |  |  |  |
| 8 | rs2102360 | | 41807985 | G | ADD | 6.87E-05 |  |  |  |  |  |  |  |  |
| 8 | rs2102360 | | 41807985 | G | DOMDEV | 5.22E-05 |  |  |  |  |  |  |  |  |
| **MCI: T-tau** | | |  |  |  |  |  |  |  |  |  |  |  |  |
| **CHR** | **SNP** | | **POSITION** | **A1** | **TEST** | **P-value** | **LCL^a^** | **EBV.transformed.LCL_Cis_JPT ^b^** | **EBV.transformed.LCL_Cis_CHB.JPT ^b^** | **EBV.transformed.LCL_Cis_CEU.CHB.JPT.YRI ^b^** | **HumanBrainTissue_pons ^c^** | **HumanBrainTissue_crblm ^c^** | **HumanBrainTissue_fctx ^c^** | **HumanBrainTissue_tctx ^c^** |
| 10 | rs2397395 | | 132617591 | A | DOMDEV | 6.45E-05 |  |  |  |  |  |  |  |  |
| 11 | rs7950171 | | 3661280 | C | GENO_2DF | 5.82E-05 |  |  |  |  |  |  |  |  |
| 14 | rs8006663 | | 68240756 | A | GENO_2DF | 4.30E-05 |  |  |  |  |  |  |  |  |
| 15 | rs12440667 | | 72018492 | T | DOMDEV | 4.84E-05 |  |  |  |  |  |  |  |  |
| 15 | rs12440667 | | 72018492 | T | GENO_2DF | 8.60E-05 |  |  |  |  |  |  |  |  |
| 1 | rs9442372 | | 1008567 | A | DOMDEV | 5.80E-05 |  |  |  |  | OVOL1 |  | OVOL1 | OVOL1 |
| **MCI: P-tau_181P_** | | |  |  |  |  |  |  |  |  |  |  |  |  |
| **CHR** | | **SNP** | **POSITION** | **A1** | **TEST** | **P-value** | **LCL^a^** | **EBV.transformed.LCL_Cis_JPT ^b^** | **EBV.transformed.LCL_Cis_CHB.JPT ^b^** | **EBV.transformed.LCL_Cis_CEU.CHB.JPT.YRI ^b^** | **HumanBrainTissue_pons ^c^** | **HumanBrainTissue_crblm ^c^** | **HumanBrainTissue_fctx ^c^** | **HumanBrainTissue_tctx ^c^** |
| 17 | | rs8077638 | 1587543 | T | DOMDEV | 2.48E-05 |  |  |  |  |  |  |  |  |
| 17 | | rs8077638 | 1587543 | T | GENO_2DF | 3.64E-05 |  |  |  |  |  |  |  |  |
| 3 | | rs1553191 | 114419299 | G | GENO_2DF | 9.25E-05 |  |  |  |  |  |  |  |  |
| 3 | | rs2362824 | 37174457 | G | DOMDEV | 7.39E-05 |  |  |  |  |  |  |  |  |
| 3 | | rs2362824 | 37174457 | G | GENO_2DF | 4.29E-05 |  |  |  |  |  |  |  |  |
| 3 | | rs6789043 | 37000871 | C | DOMDEV | 3.65E-05 |  |  |  |  |  |  |  |  |
| 3 | | rs6789043 | 37000871 | C | GENO_2DF | 1.83E-05 |  |  |  |  |  |  |  |  |
| 3 | | rs7632108 | 37233067 | A | DOMDEV | 9.88E-06 |  |  |  |  |  |  |  |  |
| 3 | | rs7632108 | 37233067 | A | GENO_2DF | 3.40E-06 |  |  |  |  |  |  |  |  |
| 7 | | rs17166357 | 94027206 | G | GENO_2DF | 4.19E-05 |  |  |  |  |  |  |  |  |
| 9 | | rs2273770 | 76945289 | T | GENO_2DF | 6.54E-05 | ostf1, C9orf95 |  |  |  |  |  |  |  |
| **AD: Aβ_1-42_** | |  |  |  |  |  |  |  |  |  |  |  |  |  |
| **CHR** | | **SNP** | **POSITION** | **A1** | **TEST** | **P-value** | **LCL^a^** | **EBV.transformed.LCL_Cis_JPT ^b^** | **EBV.transformed.LCL_Cis_CHB.JPT ^b^** | **EBV.transformed.LCL_Cis_CEU.CHB.JPT.YRI ^b^** | **HumanBrainTissue_pons ^c^** | **HumanBrainTissue_crblm ^c^** | **HumanBrainTissue_fctx ^c^** | **HumanBrainTissue_tctx ^c^** |
| 17 | | rs17794370 | 19638568 | A | GENO_2DF | 4.76E-05 |  |  |  |  |  |  |  |  |
| 17 | | rs228765 | 39541948 | G | ADD | 7.56E-05 |  |  |  |  |  |  |  |  |
| 19 | | rs735273 | 19246411 | C | DOMDEV | 2.77E-05 |  |  |  |  |  |  |  |  |
| 19 | | rs735273 | 19246411 | C | GENO_2DF | 9.02E-05 |  |  |  |  |  |  |  |  |
| 4 | | rs3796637 | 140201368 | C | ADD | 7.15E-05 |  |  |  |  |  |  |  |  |
| 4 | | rs3796637 | 140201368 | C | DOMDEV | 2.96E-05 |  |  |  |  |  |  |  |  |
| 4 | | rs3796637 | 140201368 | C | GENO_2DF | 2.18E-05 |  |  |  |  |  |  |  |  |
| 5 | | rs4702086 | 15643727 | T | DOMDEV | 9.85E-05 |  |  |  |  |  |  |  |  |
| 6 | | rs1209049 | 73326765 | A | GENO_2DF | 3.50E-05 |  |  |  |  |  |  |  |  |
| 6 | | rs9346979 | 164229469 | T | DOMDEV | 3.84E-05 |  |  |  |  |  |  |  |  |
| 6 | | rs9346979 | 164229469 | T | GENO_2DF | 8.86E-05 |  |  |  |  |  |  |  |  |
| **AD: T-tau** | |  |  |  |  |  |  |  |  |  |  |  |  |  |
| **CHR** | | **SNP** | **POSITION** | **A1** | **TEST** | **P-value** | **LCL^a^** | **EBV.transformed.LCL_Cis_JPT ^b^** | **EBV.transformed.LCL_Cis_CHB.JPT ^b^** | **EBV.transformed.LCL_Cis_CEU.CHB.JPT.YRI ^b^** | **HumanBrainTissue_pons ^c^** | **HumanBrainTissue_crblm ^c^** | **HumanBrainTissue_fctx ^c^** | **HumanBrainTissue_tctx ^c^** |
| 1 | | rs1954173 | 159944408 | G | DOMDEV | 6.30E-05 |  |  | FREB | FREB |  |  |  |  |
| 2 | | rs13026048 | 159854423 | A | GENO_2DF | 2.48E-05 |  | FLJ36175 |  |  |  |  |  |  |
| 3 | | rs2979307 | 126792264 | C | DOMDEV | 2.42E-06 |  |  |  |  |  |  |  |  |
| 3 | | rs2979307 | 126792264 | C | GENO_2DF | 6.24E-06 |  |  |  |  |  |  |  |  |
| 6 | | rs1741740 | 26536821 | A | ADD | 4.76E-05 |  |  |  |  |  |  |  |  |
| 6 | | rs2395309 | 33134224 | G | ADD | 1.78E-05 | HLA-DPA1, HLA-DPB1 |  |  |  |  |  |  |  |
| 6 | | rs2395309 | 33134224 | G | GENO_2DF | 5.57E-05 | HLA-DPA1, HLA-DPB1 |  |  |  |  |  |  |  |
| 6 | | rs9469341 | 33143855 | G | ADD | 1.66E-05 |  |  |  |  |  |  |  |  |
| 6 | | rs9469341 | 33143855 | G | GENO_2DF | 5.24E-05 |  |  |  |  |  |  |  |  |
| 7 | | rs2160093 | 20917021 | C | ADD | 3.45E-05 |  |  |  |  |  |  |  |  |
| **AD: P-tau_181P_** | | |  |  |  |  |  |  |  |  |  |  |  |  |
| **CHR** | **SNP** | | **POSITION** | **A1** | **TEST** | **P-value** | **LCL^a^** | **EBV.transformed.LCL_Cis_JPT ^b^** | **EBV.transformed.LCL_Cis_CHB.JPT ^b^** | **EBV.transformed.LCL_Cis_CEU.CHB.JPT.YRI ^b^** | **HumanBrainTissue_pons ^c^** | **HumanBrainTissue_crblm ^c^** | **HumanBrainTissue_fctx ^c^** | **HumanBrainTissue_tctx ^c^** |
| 11 | rs3818055 | | 32395329 | A | ADD | 9.22E-05 |  |  |  |  |  |  |  |  |
| 16 | rs948705 | | 55906847 | C | ADD | 1.47E-05 |  |  |  |  |  |  |  |  |
| 16 | rs948705 | | 55906847 | C | DOMDEV | 1.93E-06 |  |  |  |  |  |  |  |  |
| 16 | rs948705 | | 55906847 | C | GENO_2DF | 5.01E-06 |  |  |  |  |  |  |  |  |
| 3 | rs2979307 | | 126792264 | C | DOMDEV | 3.28E-05 |  |  |  |  |  |  |  |  |
| 3 | rs2979307 | | 126792264 | C | GENO_2DF | 9.77E-05 |  |  |  |  |  |  |  |  |
| 4 | rs10516501 | | 104433739 | G | DOMDEV | 6.15E-05 |  |  |  |  |  |  |  |  |
| 5 | rs2940521 | | 176271815 | G | GENO_2DF | 7.27E-05 |  |  |  |  |  |  |  |  |
| Abbreviations: CHR, chromosome; SNP, single-nucleotide polymorphism; A1, tested minor allele; ORIGSNP, original SNP; | | | | | | | | | | | | |  |  |
| ADD, additive effects of allele dosage; GENO_2DF,a 2 df joint test of both additive and dominance; | | | | | | | | | |  |  |  |  |  |
| DOMDEV, dominance deviation from additivity, rather specifying that a particular allele is dominant or recessive; | | | | | | | | | | | |  |  |  |
| LCL, Lymphoblastoid cell lines; EBV.transformed.LCL_Cis, Epstein-Barr virus–transformed lymphoblastoid cell lines(LCL), | | | | | | | | | | | | |  |  |
| cis-regulatory variation in the human genome (Europeans(CEU), Yoruban Africans(YRI), Han Chinese(CHB), and Japanese(JPT)); | | | | | | | | | | | | | |  |
| crblm, cerebellum; fctx, frontal cortex; tctx, temporal cortex | | | | | | |  |  |  |  |  |  |  |  |
| ^a^The type of tissues used to study SNP variation on gene expression from Dixon et al. | | | | | | | |  |  |  |  |  |  |  |
| ^b^The type of tissue used to study SNP variation on gene expression from Stranger et al. | | | | | | | | |  |  |  |  |  |  |
| ^c^The type of tissue used to study SNP variation on gene expression from Gibbs et al. | | | | | | | |  |  |  |  |  |  |  |
